# Phenotype plasticity and altered sensitivity to chemotherapeutic agents in aggressive prostate cancer cells

**DOI:** 10.3389/fcell.2023.1285372

**Published:** 2023-11-16

**Authors:** Allan I. Paxson, Loren H. Chang, Jaime M. C. Gard, William L. Harryman, Colin S. Nelson, Stella B. Salmon, Kendra D. Marr, Leah M. Wachsmuth, Anita Ramanathan, Jing Ran, Abhijeet Kapoor, Juan J. Marugan, Mark J. Henderson, Tino W. Sanchez, Anne E. Cress

**Affiliations:** ^1^ Partnership for Native American Cancer Prevention, University of Arizona, Tucson, AZ, United States; ^2^ University of Arizona Cancer Center, University of Arizona, Tucson, AZ, United States; ^3^ National Center for Advancing Translational Sciences, National Institutes of Health, Rockville, MD, United States; ^4^ Medical Scientist Training MD/PhD Program, College of Medicine Tucson, University of Arizona, Tucson, AZ, United States; ^5^ Department of Cellular and Molecular Medicine, University of Arizona, Tucson, AZ, United States

**Keywords:** prostate cancer, aggressive, chemoresistance, sensitivity, cell viability

## Abstract

In 2023, approximately 288,300 new diagnoses of prostate cancer will occur, with 34,700 disease-related deaths. Death from prostate cancer is associated with metastasis, enabled by progression of tumor phenotypes and successful extracapsular extension to reach Batson’s venous plexus, a specific route to the spine and brain. Using a mouse-human tumor xenograft model, we isolated an aggressive muscle invasive cell population of prostate cancer, called DU145^J7^ with a distinct biophysical phenotype, elevated histone H3K27, and increased matrix metalloproteinase 14 expression as compared to the non-aggressive parent cell population called DU145^WT^. Our goal was to determine the sensitivities to known chemotherapeutic agents of the aggressive cells as compared to the parent population. High-throughput screening was performed with 5,578 compounds, comprising of approved and investigational drugs for oncology. Eleven compounds were selected for additional testing, which revealed that vorinostat, 5-azacitidine, and fimepinostat (epigenetic inhibitors) showed 2.6-to-7.5-fold increases in lethality for the aggressive prostate cancer cell population as compared to the parent, as judged by the concentration of drug to inhibit 50% cell growth (IC_50_). On the other hand, the DU145^J7^ cells were 2.2-to-4.0-fold resistant to mitoxantrone, daunorubicin, and gimatecan (topoisomerase inhibitors) as compared to DU145^WT^. No differences in sensitivities between cell populations were found for docetaxel or pirarubicin. The increased sensitivity of DU145^J7^ prostate cancer cells to chromatin modifying agents suggests a therapeutic vulnerability occurs after tumor cells invade into and through muscle. Future work will determine which epigenetic modifiers and what combinations will be most effective to eradicate early aggressive tumor populations.

## 1 Introduction

Lethal metastatic prostate cancer (PCa) is defined by tumor that extends past the prostate gland. Tumor extension requires traversal through a smooth muscle capsule to reach the Batson’s plexus for systemic dissemination to the vertebral column and other metastatic sites (Harryman et al., 2016; Harryman et al., 2021). Smooth muscle invasion exposes the tumor population to a contractile tumor microenvironment, resulting in damage to the cell nuclear envelope (Lammerding and Wolf, 2016). Model studies have shown the nuclear deformation in single cells results in subsequent alterations in DNA packaging into heterochromatin, DNA damage, and DNA replication stress (Hsia et al., 2022). In PCa, nuclear envelope deformations are common in human tissue specimens from both radical prostatectomy samples and within needle biopsy specimens and are linked to metastasis (Reis-Sobreiro et al., 2018; Wang et al., 2020).

In this work, we utilized a tumor cell model that had invaded and traversed through a contractile smooth muscle barrier, which was retrieved from a mouse xenograft model pioneered by us to study early invasion events (McCandless et al., 1997). Our previous work has shown that biophysical cell adhesion phenotypes can markedly affect tumor invasion properties (Rubenstein et al., 2019) and contribute to drug resistance (Damiano et al., 1999). We therefore characterized the cell adhesion biophysical phenotype and developed a high-throughput screening assay to examine the sensitivity to known chemotherapeutic agents between the two populations of tumor. This study reports six therapeutic compounds that exhibit increased potency against the aggressive DU145^J7^ PCa cell line, three that exhibit increased potency against parental DU145^WT^ PCa cell line, and two compounds that have no significant difference between the tumor populations in survival response.

## 2 Materials and methods

### 2.1 Cell culture and mouse xenograft model

DU145^WT^ cells were obtained from American Type Culture Collection, ATCC, (Manassas, VA). DU145^J7^ cells were derived from DU145^WT^ cells via three 5-week serial *in vivo* passages through a NSG mouse diaphragm. Briefly, human DU145^WT^ PCa cells gained access to the inferior surface of the mouse diaphragm by an intraperitoneal injection route. Once injected, mice were cared for by the University of Arizona Experimental Mouse Shared Resource (EMSR) core. Mice were monitored for up to 6 weeks after injection and any animal showing signs of tumor metastasis (weight loss, ruffled fur, and signs of pain) was removed from the study and the resulting diaphragm was harvested for retrieval of tumor cells on the superior surface. Microscopic imaging of tumor on the inferior surface, within the smooth muscle diaphragm, or on the superior surface confirmed muscle invasion. After the third passage through the diaphragm of the mouse xenograft model, tumor cells present on the superior surface were collected and prepared for cell culture. Both cell populations–DU145^WT^ and DU145^J7^—were cultured in Iscove’s DMEM (Corning, Salt Lake City, UT) with 10% Fetal Bovine Serum (Omega Scientific Inc., Tarzana, CA.) using standard tissue culture conditions. The experimental mouse studies were reviewed and approved by the Institutional Animal Care and Use Committee as Protocol Number: 07029. The protocol was conducted in accordance with all applicable federal and institutional policies, procedures, and regulations, including the PHS Policy on Humane Care and Use of Laboratory Animals, USDA regulations (9 CFR Parts 1, 2, 3), the Federal Animal Welfare Act (7USC 2131 et. Seq.), the Guide for the Care and Use of Laboratory Animals, and all relevant institutional regulations and policies regarding animal care and use at the University of Arizona.

### 2.2 STR analysis

The Arizona Genetics Core (University of Arizona, Tucson, AZ; Facility RRID:SCR_012429) provided cell line authentication analysis using short tandem repeats (STR) to confirm the DU145^J7^ was derived from the DU145^WT^ parental population.

### 2.3 ECIS measurements

Electrical properties of confluent or wounded epithelial monolayers were measured using electric cell–substrate impedance sensing (ECIS) (Rubenstein et al., 2019). Cell adhesion characteristics were based on changes in resistance/capacitance measurements every 4 min to current flow applied at different frequencies (Applied Biophysics) during optimal culture conditions. The 8-well chamber slide with ten 250 μm electrodes (Applied Biophysics, 8W10E + PET) was first stabilized by coating with 10 mM cysteine in water and then rinsed twice with sterile distilled water. The 8-well chamber slide was coated with laminin at 37°C for 1 h, cells were added at 200,000 cells per well in 400 µL media in duplicates, and resistance/capacitance was measured at 4,000 Hz as previously reported (Wegener et al., 2000; Hong et al., 2011). Electric wounding of the monolayer was done 16 h after monolayer growth and the capacitance recorded in response to the wound for a total time of 48 h. Capacitance of the monolayer increases as the complex cell surface morphology and membrane folding states and protrusive activity increases (Memmel et al., 2014). For comparative purposes, all data has been normalized by the ECIS software by dividing all values by that obtained at the time listed in the zero-time box.

### 2.4 Flow cytometry

Flow cytometry analysis was used to quantitate MMP-14 expression (using the eBioscience Intracellular Fixation & Permeabilization Buffer Set, Invitrogen) and H3K27 nuclear expression (using the eBioscience FOXP3/Transcription Factor Staining Buffer Set, Invitrogen) within tumor cell suspensions. The cell lines were harvested, washed in PBS, fixed and permeabilized following the manufacturer’s appropriate recommended protocol. After a 1 h incubation at room temperature with the primary antibody, the cell suspension was washed twice with the corresponding buffer. The remaining cell pellet was resuspended in the appropriate permeabilization buffer and incubated for 1 h at room temperature with secondary antibody. Cells were then washed twice and resuspended in PBS before being analyzed by flow cytometry. Antibody dilutions for MMP14 (Abcam, ab51074) and H3K27 (Cell Signaling Technology, C36B11) primary antibodies were 1:200 and 1:500 dilutions respectively. The secondary antibody used in both samples was anti-rabbit at 1:500 dilution.

### 2.5 High-throughput screen for cell viability

In a quantitative high-throughput screen for cell viability (qHTS), 5 µL of DU145^WT^ or DU145^J7^ cells in suspension (1,000 cells/well) were dispensed into 1,536-well white walled tissue culture treated plates using a Multidrop Combi Reagent Dispenser (Thermo Fisher Scientific, Waltham, MA). The subsequent steps of the viability screen were performed on a fully integrated robotic system (Kalypsys, San Diego, CA) containing one RX-130 and two RX-90 anthropomorphic robotic arms (Staübli, Duncan, SC). 20 nL of compound in 7-point, 3-fold serial dilutions were transferred to the assay plates using the 1,536-well Kalypsys pintool system. The National Center for Advancing Translational Science (NCATS) Pharmaceutical collection of approved drugs (Huang et al., 2019) and the Mechanistic Interrogation Plate collection of approved and investigational oncology agents (Mathews Griner et al., 2014) were selected for and tested. After 48 h of incubation at 37°C and 5% CO_2_, 5 µL of CellTiter-Glo (Promega Corp., Madison, WI) was added to each well. Following a 10-min incubation at room temperature, luminescence was measured on the ViewLux uHTS Microplate Imager (PerkinElmer, Waltham, MA) with 2 s exposure and clear filters. For follow-up screening, 20 nL of compound in 11-point, 3-fold serial dilutions were acoustically dispensed from compound plates using an Echo 550 Acoustic Liquid Handler (Labcyte Inc., San Jose, CA). After 48 h of incubation at 37°C and 5% CO_2_, 5 µL of CellTiter-Glo was added to each well and plates were shaken on the Multidrop for 5 min. Following a 10-min incubation at room temperature, luminescence was measured on ViewLux. Each plate contained four columns of controls: no cells (background control), DMSO only (vehicle control), and bortezomib as well as taxotere/docetaxel in duplicate 16-point dose response, respectively, as positive controls. Raw luminescence results were normalized to DMSO (neutral control) and no cells (maximum response). Dose response curves were fitted using the Hill equation based on a previously established qHTS data analysis workflow (Inglese et al., 2006; Huang et al., 2022).

### 2.6 Compound selection and dilutions

Compounds selected for additional studies were purchased from various manufacturers* and all dissolved in DMSO. A 10-point serial dilution was performed to generate a range of concentrations. Compounds were each prepared at different maximum concentrations and diluted with either a dilution factor of two or three. All dilutions were prepared in Iscove’s DMEM (Corning, Salt Lake City, UT) with 10% Fetal Bovine Serum (Omega Scientific Inc., Tarzana, CA). Determination of the optimized concentration range for each compound was based on published values for compound’s effect on DU145^WT^ cells or from NCATS preliminary screen with both the parental and aggressive cell population.

*Compound Manufacturers: Vorinostat (Cell Signaling Technology, Danvers, MA). 5-Azacitidine (Sigma-Aldrich, Burlington, MA). Bleomycin (Thermo Scientific Chemicals, Waltham, MA). Fimepinostat (Medchemexpress, LLC, Monmouth Junction, NJ). Bortezomib (Targetmol Chemicals Scientific, Inc., Boston, MA). Olaparib (Sigma, St. Louis, MO). Mitoxantrone (Sigma-Aldrich, Burlington, MA). Daunorubicin (Sigma-Aldrich, Burlington, MA). Gimatecan (Targetmol Chemicals, Inc., Boston, MA). Docetaxel (Sigma-Aldrich, Burlington, MA). Pirarubicin (Sigma-Aldrich, Burlington, MA).

### 2.7 Cell viability assay

Analysis of cell viability was conducted with CellTiter-Glo Luminescent Cell Viability Assay (Promega Corp., Madison, WI) according to manufacturer protocols. DU145^WT^ and DU145^J7^ cells were plated at 9×10^3^ cells/well in a black 96-well plate with a clear bottom and allowed to attach for 24 h in incubation. After 24 h, culture media was replaced with 100 µL of each compound at different increasing concentrations and incubated for 48 h. After 48 h, 100 µL of CellTiter-Glo reagent was added to each well, without media replacement. The 96-well plate was then placed on a titer plate shaker for 10 min followed by plate reader analysis using 1s integration time per well in a Gen5 Microplate Reader v.2.01.14 (BioTek Instruments, Inc., Winooski, VT). Survival curves were generated using GraphPad Prism v.10.0.1 (218).

### 2.8 IC_50_ determination

IC_50_ values were estimated directly from the survival curves generated in GraphPad Prism. Averages from replicates were obtained with standard deviations using Microsoft Excel (Supplementary Table S1).

### 2.9 Statistical analysis

CellTiter-Glo Luminescent Cell Viability Assay was performed with at least two biological replicates representing four technical replicates for each tested cell line. Error bars in the survival curve represent standard deviation from the mean. ECIS data error bars represent standard deviation. Standard deviation error bars for ECIS measurements were provided by the ECIS software (Applied Biophysics), v.1.4.18.0 PC, software updated February 2023. Statistics for flow cytometry analysis are provided through the Attune™ NxT Software v4.2.0.

## 3 Results

### 3.1 Isolation and characterization of the DU145^J7^ cell population

**FIGURE 1:**
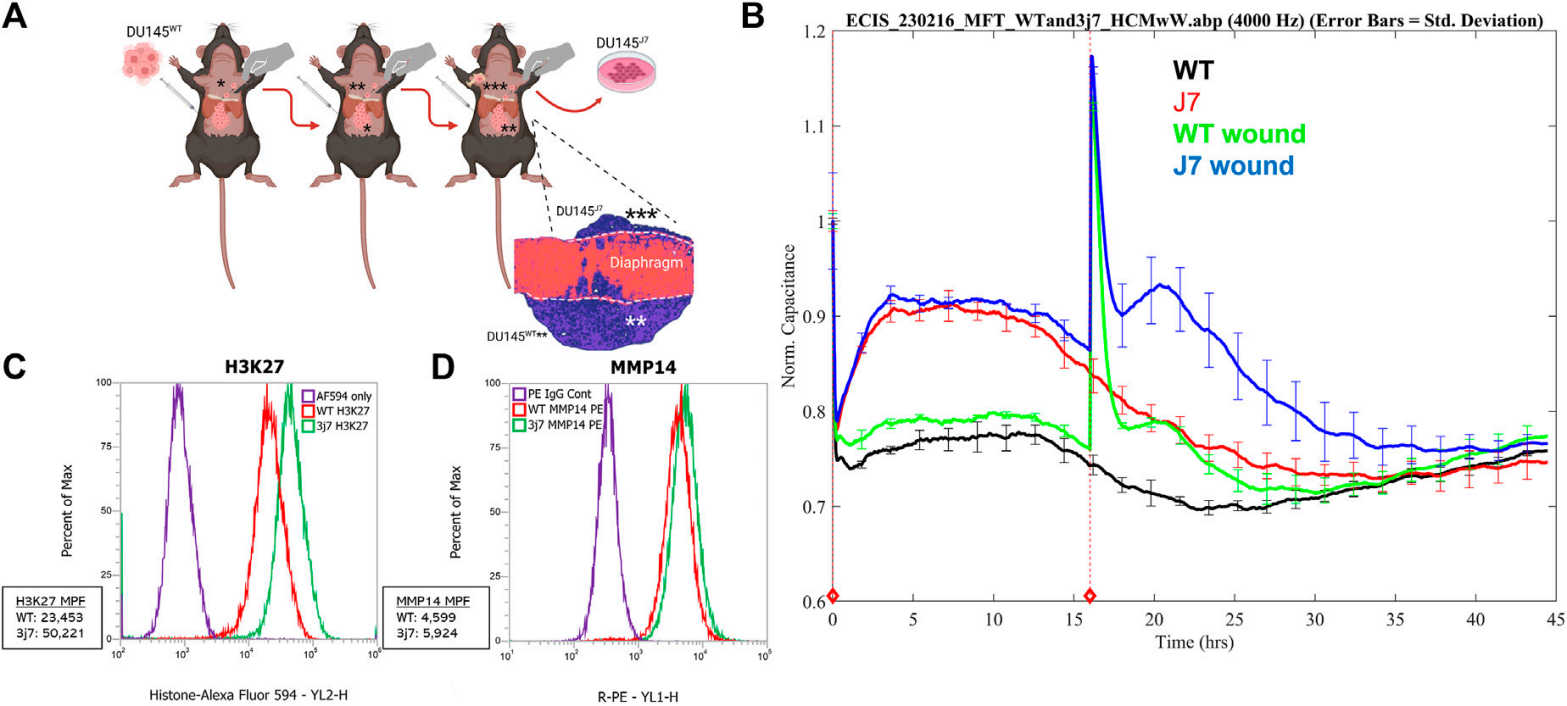
**(A–D)** *In vivo* retrieval and characterization of DU145^J7^cell sub-population. **(A)** DU145^WT^ parent cells were seeded onto the inferior side of the mouse diaphragm. After traversing the muscle layer, the successful cells were retrieved from the superior surface 5 weeks later and grown under standard cell culture conditions. The resulting population (*) was re-injected into mice and this was repeated two more times via serial passage and the resulting population is called DU145^J7^ (depicted as population ***). Figure created with Biorender. **(B)** Electric cell-substrate impedance sensing of capacitance at 4,000 Hz of unwounded monolayers of DU145^WT^ (WT, black line), DU145^J7^ (J7, red line), wounded monolayers of DU145^WT^ (WT wd, green line), and wounded monolayers of DU145^J7^ (J7 wd, blue line). The biophysical normalized capacitance measurements were recorded every 4 min over 48-h with wounding occurring at 16-h (red vertical line). **(C)** Flow cytometry analysis of H3K27 in the DU145^WT^ (WT, red line); Mean Peak Fluorescence (MPF): 23,453, and DU145^J7^ (3j7, green line) (MPF: 50,221). PE IgG used as control (purple line). Measurements represent 30,000 events. **(D)** Flow cytometry analysis of MMP14 in the DU145^WT^ (WT, red line) (MPF: 4,599) and DU145^J7^ (3j7, green line) (5,924). AF594 used as control (purple line). Measurements represent 30,000 events.

DU145^WT^ tumor cells are an epithelial population of human PCa cells with heterogeneous adhesion properties (Pollan et al., 2021). In a xenograft mouse model, DU145^WT^ cells will form tumors, seed onto the inferior surface of the smooth muscle diaphragm, and invade into the diaphragm muscle of the animal but will not metastasize (Harryman et al., 2022). Therefore, after IP injection and 5 weeks of incubation in the mouse, DU145^WT^ tumor cells that had invaded and migrated through the inferior smooth muscle diaphragm of the mouse to reach the superior surface were collected and passaged again *in vivo* (schematized, Figure 1A). This was done three times, and the resulting metastasis-capable cell subpopulation is called DU145^J7^ (Figure 1A, histology inset). Short tandem repeats (STR) confirmed that the DU145^J7^ population was derived from the parental DU145^WT^ population. While related, these two tumor cell populations proved to be different in their biophysical characteristics as judged first by electric cell–substrate impedance sensing (ECIS) (Figure 1B). ECIS, a non-invasive population measurement of capacitance, measures a live cell monolayer in real time for 48-h under ideal growth conditions. Increased capacitance of both cell populations during the first 15 h signifies cell proliferation (Figure 1B). In the DU145^WT^ population, the normalized capacitance value stabilized at approximately 0.8, followed by an increase in capacitance in response to the wounding event at 16 h. Recovery of the DU145^WT^ cell population to pre-wound levels was reached at 10 h after wounding. In contrast, the DU145^J7^ population had a higher initial capacitance at 0.9 followed by an increase after wounding which persisted with recovery to pre-wounding levels occurring by 21 h after wounding. The increased capacitance response of the DU145^J7^ cells to a higher level over a longer period indicates a higher protrusive activity of the population, characteristic of aggressive tumor cells (Memmel et al., 2014). In addition to biophysical characteristic differences between the cell lines, the DU145^J7^ population increases H3K27 histone population by 2-fold (Mean Peak Fluorescence (MPF) DU145^WT^: 23,453 and MPF DU145^J7^: 50,221) as determined by flow cytometry (Figure 1C). Another marker of aggressive tumor cells is the expression of matrix metalloproteinase 14 (MMP14). MMP14 expression in the wild-type cell line has an MPF of 4,599 whereas the aggressive variant has an MPF of 5,924, a 1.3-fold increase (Figure 1D). Histone upregulation increases epigenetic modifications promoting tumor suppression in cancers (Hsia et al., 2022). Likewise, increased presence of MMP14 stimulates infiltration of surrounding tissues for tumor progression (Conlon and Murray, 2019).

### 3.2 Aggressive PCa cells display therapeutic vulnerability to epigenetic modulators

**FIGURE 2:**
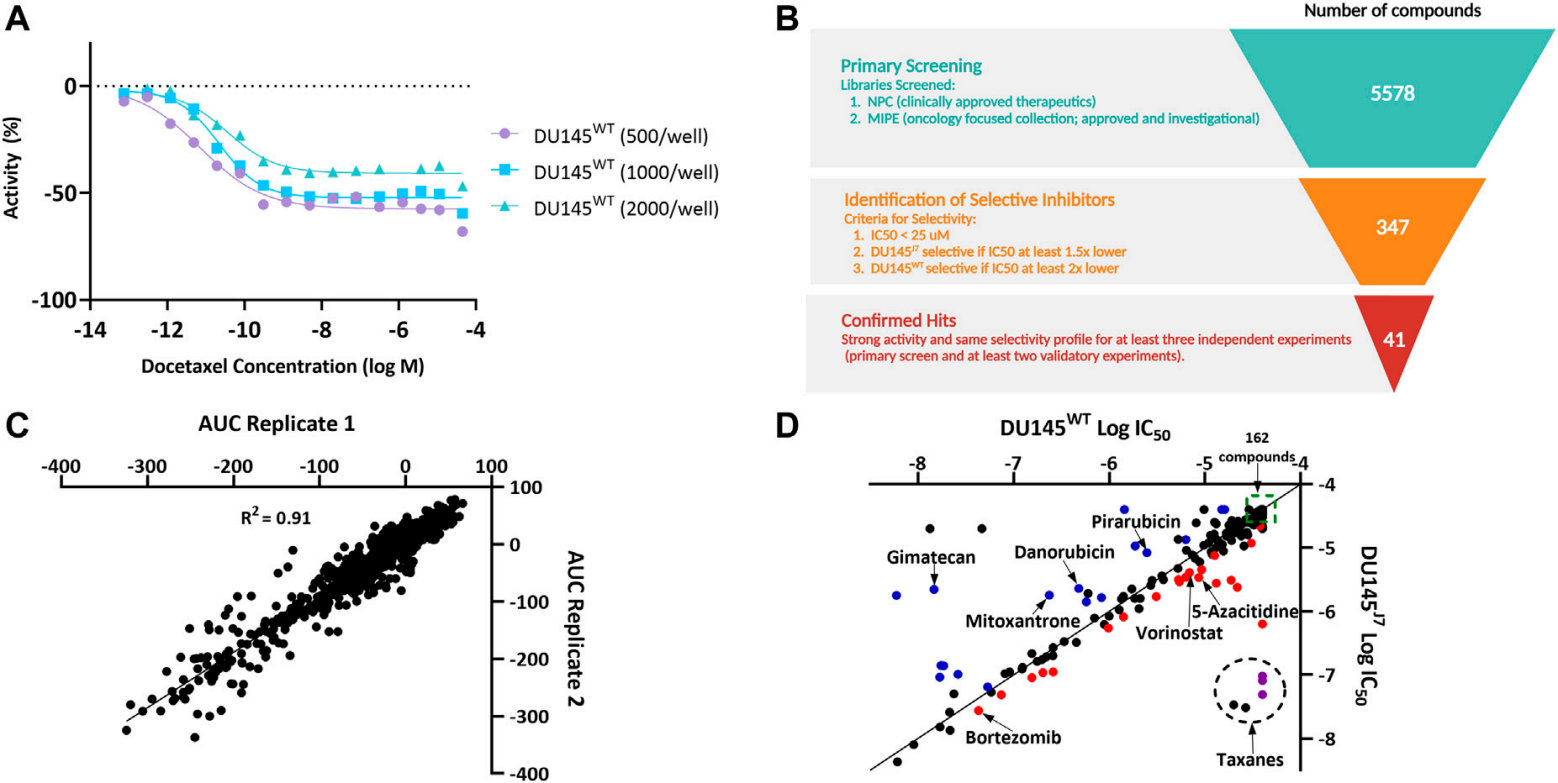
**(A–D)**. High-throughput screening of NCATS compound libraries on DU145^WT^ and DU145^J7^. **(A)** Dose response curves demonstrating the optimization of DU145 cell density for the 1,536-well cell viability assay using a docetaxel titration. **(B)** Schematic summarizing the high-throughput screening selection process that led to the identification of 41 confirmed selective inhibitors. Figure created in Biorender. **(C)** Plot of DU145^WT^ and DU145^J7^ area under the curve (AUC) values from two replicates for the 347 compounds selected for follow up. **(D)** Plot showing selectivity of the 347 compounds. The data are average values of at least two independent experiments, with each containing at least two technical replicates. Compounds deemed selective based on both IC_50_ fold-shift and manual inspection of dose response curves are labeled in blue (DU145^WT^-selective), red (DU145^J7^-selective), or purple (DU145^J7^-selective taxanes). Compounds in the larger dotted circle denote taxanes. 162 compounds (clustered in the green box) showed no activity in either cell line.

A miniaturized cell viability assay using CellTiter-Glo was developed to identify compounds with selective effects on the DU145^WT^ or the DU145^J7^ line (Supplementary Figure S1A). Optimization of the qHTS assay involved using varying cell densities of DU145 cells and a serial dose response treatment with docetaxel, the current standard of treatment, in 1,536-well tissue culture-treated plates (Figure 2A, Supplementary Figure S1B). Based on efficacy and curve fit, 1,000 cells per well was selected as the optimal cell density. A primary screen of 5,578 compounds from the NCATS Pharmaceutical Collection (NPC) and the Mechanistic Interrogation Plate (MIPE) libraries were screened in 7-point dose response. 347 compounds that showed activity (IC_50_ < 25 µM) and selectivity (IC_50_ at least 1.5x lower in DU145^J7^ than in DU145^WT^, or at least 2x lower in DU145^WT^ than in DU145^J7^) were identified as potential selective inhibitors (Figure 2B). The median Z-factor for plates containing DU145^WT^ or DU145^J7^ was 0.59 and 0.61, respectively, demonstrating the high quality of the qHTS assay (Supplementary Figure S1C). In a follow-up screen, all 347 compounds were retested in an 11-point dose response, and area under the curve (AUC) values of two replicates were plotted for both DU145^WT^ and DU145^J7^ (Figure 2C). A high correlation measured by linear regression (*R* ^2^ = 0.91) was observed between experiments, demonstrating a high degree of replicability and reproducibility of the assay. 41 compounds were confirmed as selective inhibitors (Figure 2D) for DU145^J7^ (red or purple circles) or DU145^WT^ (blue) based on both IC_50_-fold shift and manual inspection of the dose response curve. Seven compounds, including 5-azacitidine (Supplementary Figure S1E) were prioritized for further validation.

**FIGURE 3:**
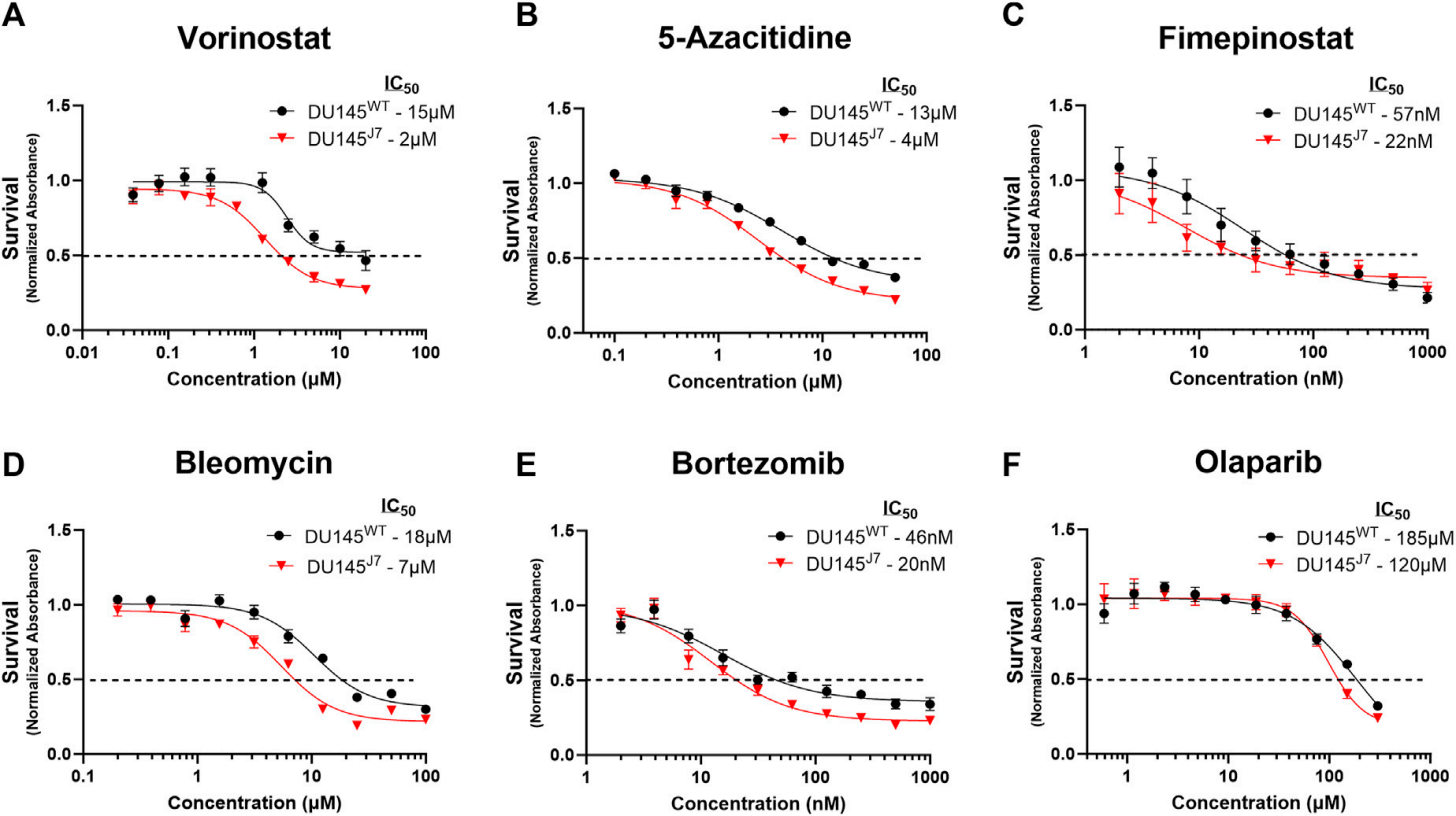
**(A–F)** DU145^J7^ are sensitive primarily to epigenetic modifiers. Dose response curves of compounds demonstrating significantly increased killing activity against aggressive DU145^J7^ (red triangles) compared to DU145^WT^ (black squares), *in vitro*. A luminescent cell viability assay was used to determine the sensitivity to treatment with increasing concentrations of anti-cancer drugs. Points represent average values of technical replicates and error bars represent standard deviation for each data point. All figures represent 1 of at least 2 biological replicates with 4 technical replicates each. The inhibitory concentration for 50% of cell growth (IC_50_) values were estimated directly by intersection of the 0.5 absorbance value and the concentration (dotted horizontal line).

In DU145^WT^ and DU145^J7^ cells, 48-h treatment with either vorinostat, 5-azacitidine, bleomycin, fimepinostat, olaparib, or bortezomib reveals therapeutic vulnerabilities in the DU145^J7^ cell population. Vorinostat, an inhibitor of class I, II, and IV histone deacetylases (iHDAC), had an IC_50_ of 15 µM in the DU145^WT^ cell population as compared to 2 µM in DU145^J7^, a 7.5-fold difference between IC_50_ values in the cell populations (Figure 3A). 5-azacitidine, an inhibitor of DNA-methyltransferase (iDNMT), also showed altered sensitivities between the two populations, with a 3.25-fold difference in IC_50_ (IC_50_ DU145^WT^: 13 µM and IC_50_ DU145^J7^: 4 µM) (Figure 3B). Fimepinostat, a dual-action class I and II HDAC inhibitor and Phosphoinositide 3-kinase inhibitor (iPI3K), had greater potency against DU145^J7^ as compared to DU145^WT^ with an IC_50_ value of 22 and 57 nM for the aggressive and wild-type cell-populations respectively (Figure 3C). Bleomycin, an inducer of DNA double-stranded breaks, had an IC_50_ value of 18 µM in the wild-type population and 7 µM in the aggressive cell population (Figure 3D). Lastly, bortezomib, a proteasome inhibitor, and olaparib, an inhibitor of poly (ADP-ribose) polymerase (iPARP), had similar almost 2-fold differences between IC_50_ values in the DU145^WT^ and DU145^J7^ cell populations (bortezomib DU145^WT^: 46 nM and DU145^J7^: 20 nM) (Figure 3E) (olaparib DU145^WT^: 185 µM and DU145^J7^: 120 µM) (Figure 3F). (See Supplementary Table S1 for averaged IC_50_ values from biological replicates presented in Supplementary Figure S3, S4).

### 3.3 Aggressive PCa cells develop resistance to topoisomerase inhibitors

**FIGURE 4:**
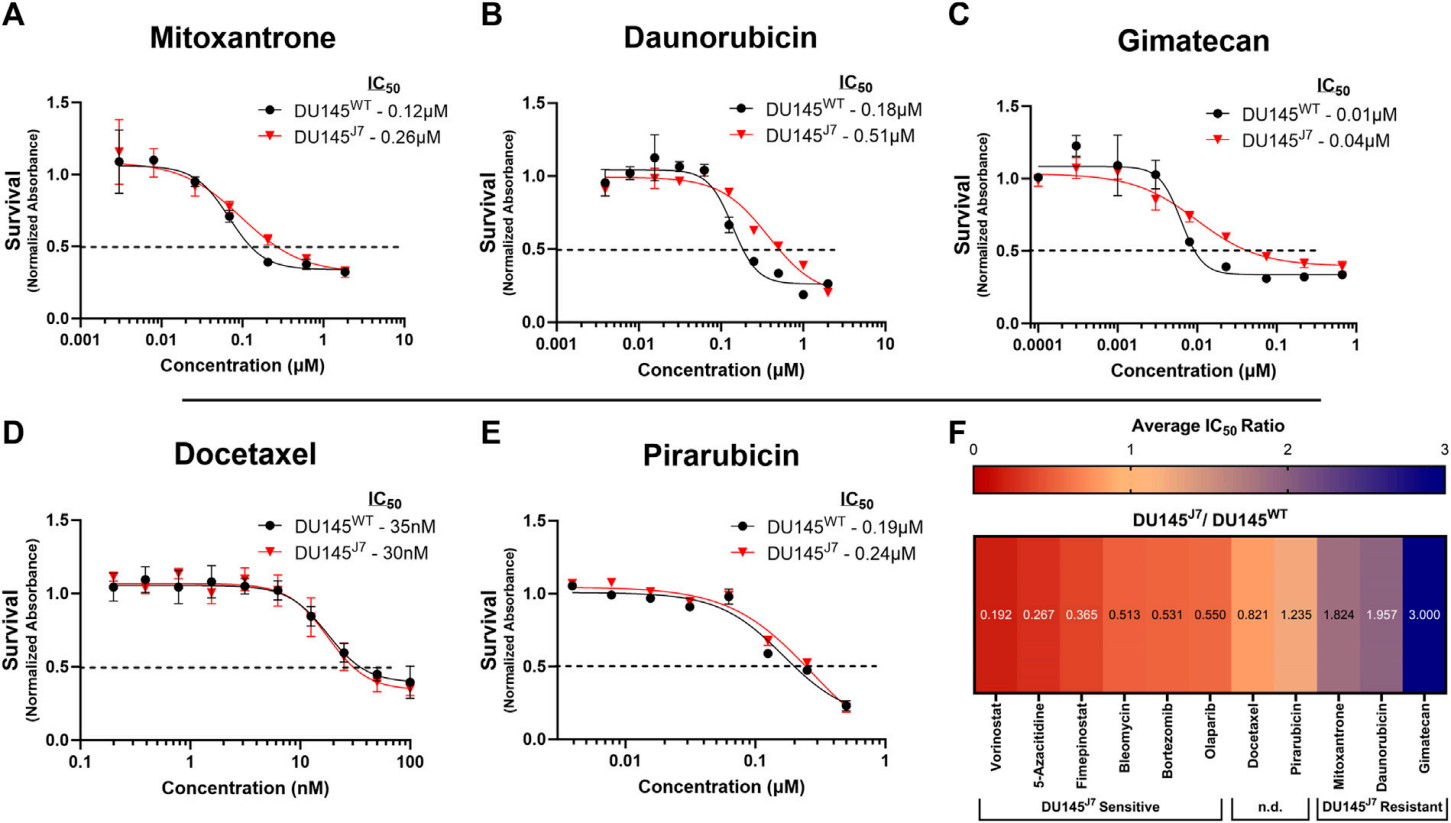
**(A–F)** DU145^J7^ resistance to topoisomerase inhibitors; two agents that lack a differential response; heat map of cell line response to anti-cancer compounds. **(A–E)** Survival curves for increasing concentrations of selected agents in DU145^J7^ (red triangles) compared to DU145^WT^ (black squares), *in vitro*. The data are average values of technical replicates and error bars represent one standard deviation for each data point. All figures represent 1 of at least 2 biological replicates with 4 technical replicates of each group. The inhibitory concentration for 50% of cell growth (IC_50_) values were estimated directly by intersection of the 0.5 absorbance value and the concentration (dotted horizontal line). **(F)** Heat map represents the ratio of each IC_50_ value (Supplementary Table S1) for aggressive DU145^J7^ population relative to the DU145^WT^ parental population. Ratio values < 1 indicate DU145^J7^ sensitivity to a compound while numbers >1 indicate DU145^J7^ resistance to a compound. For purposes of this study, values equal to 1.0 ± 0.25 were considered to have no significant difference (n.d.) in response to the compounds.

The DU145^J7^ cell population as compared to the DU145^WT^ was resistant to topoisomerase I or II inhibitors (iTopoI or iTopoII). Using the criteria of a minimum approximate two-fold difference in IC_50_ values, mitoxantrone (iTopoII), daunorubicin (iTopoII), and gimatecan (iTopoI) showed greater potency against the parental cell line as compared to the aggressive variant. Treatment with mitoxantrone generated an IC_50_ value of 0.12 µM for the DU145^WT^ cell population whereas the IC_50_ value for DU145^J7^ cells was 0.26 µM (Figure 4A). Similarly, daunorubicin had a 2.83-fold difference in IC_50_ between the tested cell populations (IC_50_-DU145^WT^: 0.18 µM and IC_50_-DU145^J7^: 0.51 µM) (Figure 4B). The difference in sensitivity was largest for gimatecan, which showed a 4-fold difference between IC_50_ value (IC_50_-DU145^WT^: 0.04 µM and IC_50_-DU145^J7^: 0.01 µM) (Figure 4C). In summary, these results suggest a DU145^J7^ resistance to Topoisomerase I inhibitors. (*See* Supplementary Table S1 for averaged IC_50_ values from biological replicates presented in Supplementary Figure S5).

### 3.4 Current standard of treatment, docetaxel, and a newly reported topoisomerase II inhibitor, pirarubicin, have similar survival response in both cancer cell types

Docetaxel is one of the current chemotherapy treatments for PCa (Sekhoacha et al., 2022). When comparing the docetaxel (iTubulin) IC_50_ values between DU145^WT^ and DU145^J7^ there was no apparent difference in response to treatment (IC_50_-DU145^WT^: 35 nM and IC_50_-DU145^J7^: 30 nM) (Figure 4D). Likewise, a lack of significant difference in response was seen following treatment with pirarubicin (iTopoII). Pirarubicin treatment resulted in an IC_50_ value of 0.19 µM in the DU145^WT^ and an IC_50_ of 0.24 µM in the DU145^J7^ cell populations (Figure 4E) (Supplementary Table S1 for averaged IC50 values from biological replicates presented in Supplementary Figure S6).

### 3.5 Summary heat map of survival response to therapeutic agents

A summary heat map of survival responses was generated (Figure 4F) to compare the ratio of each averaged IC_50_ value (original data in Supplementary Table S1) for DU145^J7^ population relative to the DU145^WT^ parental population. Ratio values < 1 indicated DU145^J7^ sensitivity to a compound while numbers >1 indicate DU145^J7^ resistance to a compound. The data indicates that effective agents with increased sensitivity for the DU145^J7^ population include vorinostat (inhibitor of class I, II, and IV histone deacetylases), fimepinostat (a combination iHDAC and PI3K inhibitor), and bleomycin (an effective generator of hydoxyl radicals and DNA double-strand breaks). The heat map also highlights the increased resistance of the DU145^J7^ population for the topoisomerase inhibitors with IC_50_ values occurring in the µM range (Supplementary Table S1).

## 4 Discussion

Aggressive and lethal PCa is defined in part by the ability of the tumor to escape organ confinement and display extraprostatic extension (EPE). EPE of cancer is the pT3a pathologic stage of prostatic adenocarcinoma with an increased risk of biochemical recurrence, distant metastases, and lower cancer-specific survival (Cheng et al., 1999; Ball et al., 2015; Fleshner et al., 2016). This work determined if currently available chemotherapeutic agents would be preferentially active in reducing the survival of a tumor that successfully invaded and traversed smooth muscle (modeled by DU145^J7^ population) as compared to the parental population (DU145^WT^). We first characterized aggressive markers in the DU145^WT^ and DU145^J7^ cell lines using *in vitro* experiments with both cell populations. These experiments revealed an approximate 2-fold increase in histone H3K27 and a 1.3-fold increase in matrix metalloproteinase, MMP14, enzyme expression in the DU145^J7^ aggressive cell line as compared to the parental DU145^WT^ cell line. Both markers, H3K27 and MMP14, are found in aggressive PCa cells (Conlon and Murray, 2019; Hsia et al., 2022). Additionally, increased protrusive activity was measured by ECIS in the DU145^J7^ cell line as compared to the wild-type cells confirming phenotypic differences between the cell populations. After characterization, selecting compounds of interest for this study was made with a cut-off of an approximate two-fold sensitivity in the IC_50_ concentration.

Our findings suggest that a tumor population that invades and migrates through a contractile muscle gains a sensitivity to iHDACs and iDNMTs. Increased sensitivity to these inhibitors is consistent with a continued requirement of these aggressive tumor cells that navigate the contractile smooth muscle. Recent reports in a tissue culture model system revealed that confined migration of tumor cells induces heterochromatin formation, alters chromatin accessibility, and decreases transcription using a mechanism, that is, dependent upon HDACs (Hsia et al., 2022). Since confined migration occurs in PCa during EPE (i.e., muscle invasion and escape), and epigenetic remodeling is a known feature of successful metastatic PCa (Hsia et al., 2022), future work will determine which combinations of chromatin remodeling inhibitors can block early metastatic success of the aggressive tumor subpopulations.

While iHDAC and iDNMT are principally used to treat hematological cancers, including T-cell lymphoma, limited efficacy has been reported for solid tumors (Jenke et al., 2021; Liang et al., 2023). Our results suggest that muscle invasive PCa may be a particular sub-type of solid tumor that would be sensitive to these inhibitors. Namely, HDAC inhibitors vorinostat and fimepinostat show greater potency against the aggressive cell population of PCa (DU145^J7^) as compared to the parent population DU145^WT^. Since HDACs are known to be over-expressed in PCa, targeting these epigenetic modulators in the muscle invasive T3 stage of PCa could be successful in halting aggressive disease before it becomes systemic (Weichert et al., 2008).

Now, some studies suggest HDAC inhibitors may increase an epithelial-to-mesenchymal (EMT) phenotype transition in PCa treatment (Kong et al., 2012) while others suggest a change in the mesenchymal-to-epithelial transition (MET) phenotype (Wang et al., 2015). Since muscle invasive PCa is a heterogenous population of epithelial cells, with different tumor cell phenotypes appearing as a unit of epithelial-to-mesenchymal cooperation (Harryman et al., 2022), the success of HDACs as therapeutic agents may rely on understanding these complex tumor phenotypes and iHDAC mechanisms (McClure et al., 2018). This point is underscored by recent work reporting transcriptomic remodeling occurring during PCa progression as detected by single cell sequencing (Chen et al., 2021) and the persistence of specialized phenotypes associated with recurrent disease (Taavitsainen et al., 2021). We find that bleomycin, a known compound for inducing DNA-DSB (Truxova et al., 2017), exhibited a potency against the DU145^J7^ cell population. Since bleomycin induces DNA-DSB and increases oxidative stress (Truxova et al., 2017), this suggests an added vulnerability of the tumor population that has traversed the contractile, nuclear damaging environment of the muscle layer. Interrupting successful DNA-DSB repair in this aggressive PCa sub-population may suggest alternative combinatorial therapeutic agents. In this regard, the DU145^J7^ sensitivity to olaparib (Figure 3F) may show synergistic lethality with the HDAC inhibitors or bleomycin by targeting non-homologous end joining (NHEJ).

When testing the standard treatment of care for metastatic castration-resistant PCa docetaxel (Sekhoacha et al., 2022), a shift in potency when comparing cell viability in the DU145^J7^ and DU145^WT^ cell lines was not observed (Figure 4D, Supplementary Figure S1D). However, several compounds belonging to the taxane class did show apparent selectivity for DU145^J7^ (Figure 2D, purple circles), but examining the underlying dose response data revealed the IC_50_ shift between the DU145^WT^ and DU145^J7^ curves is not as significant as the selectivity plot implies. This is because many of the taxanes showed partial effects on viability below −50% activity, leading to an inability to calculate an IC_50_ value (Supplementary Figures S2A-D). Consequently, while some of the taxanes had nanomolar IC_50_ values in DU145^J7^, in some cases they didn’t reach 50% efficacy in DU145^WT^ and were classified as inactive (set to 40 µM), yielding a large apparent shift in IC_50_ (Figure 2D). Nevertheless, three taxanes (cabazitaxel, epothilone B, and ixabepilone) showed clear separation in efficacy between the cell lines and were considered selective based on their dose-response curves (Supplementary Figure S2).

Finally, less-effective agents against the DU145^J7^ population include topoisomerase inhibitors. Topoisomerase enzymes have important biological functions in maintaining proper DNA replication/transcription processes in normal cells and themselves cause DNA breaks during their mechanism of action (Pommier et al., 2016; Delgado et al., 2018). The apparent resistance to topoisomerase inhibitors, but sensitivity to inhibitors of epigenetic modifiers, may indicate a priority need for chromatin remodeling capability during successful invasion into and through the contractile muscle by the tumor sub-population. Future work will directly test the need for dynamic chromatin remodeling as a route for successful escape of prostate tumors through smooth muscle.

PCa disseminates by EPE to reach the para-vertebral Batson’s plexus -- a series of primordial valveless veins (Nathoo et al., 2011) and nerves that account for how tumor originating in the prostate organ can reach the known dissemination sites of the pelvic bones, the vertebral column, and brain (Batson, 1940). Interception of PCa dissemination at its earliest stages is now an actionable goal since EPE can be detected by multiparametric magnetic resonance imaging (mpMRI) and continued improvements for 30-min whole body scan times and AI assistance (Nakanishi et al., 2022) will enable the early detection of this sub-type of aggressive tumor. This advance coupled with our increased ability to target this sub-type of PCa will add significantly to the choices available to combat this chronic and indolent cancer.

## Data Availability

The raw data supporting the conclusion of this article will be made available by the authors, without undue reservation.
