# Supplementary material for: Phenotype plasticity and altered sensitivity to chemotherapeutic agents in aggressive prostate cancer cells
Source: Front Cell Dev Biol. 2023 Nov 16;11:1285372. doi: 10.3389/fcell.2023.1285372 (PMC10690371; doi:10.3389/fcell.2023.1285372)
Supplement: Supplementary file 1 [file DataSheet1.pdf]

Supplementary Material

1.1 Supplementary Table 1

**Supplementary Table 1.**	Average IC_{50} ± SD		
Compound	$DU145^{WT}$	$DU145^{J7}$	$DU145^{J7}/DU145^{WT}$ Ratio
*Vorinostat<br>(iHDAC)	13 ± 2.83µM	2.5 ± 0.71µM	0.192
**5-Azacitidine<br>(iDNA-methyltransferase)	15 ± 1.53µM	4.0 ± 1µM	0.267
*Fimepinostat<br>(Dual iHDAC & iPI3K)	52 ± 7.78nM	19 ± 4.95nM	0.365
**Bleomycin<br>(Induces DNA DSB)	15 ± 4.62µM	7.7 ± 3.06µM	0.513
**Bortezomib<br>(iProteasome)	49 ± 3.79nM	26 ± 9.81nM	0.531
*Olaparib<br>(iPARP)	218 ± 45.96µM	120 ± 0µM	0.550
*Docetaxel<br>(iTubulin)	28 ± 10.61nM	23 ± 10.61nM	0.821
**Pirarubicin<br>(iTopoisomerase II)	0.17 ± 0.03µM	0.21 ± 0.03µM	1.235
*Mitoxantrone<br>(iTopoisomerase II)	0.17 ± 0.06µM	0.31 ± 0.06µM	1.824
**Daunorubicin<br>(iTopoisomerase II)	0.23 ± 0.13µM	0.45 ± 0.13µM	1.957
**Gimatecan<br>(iTopoisomerase I)	0.01 ± 0µM	0.03 ± 0.01µM	3.000

Supplementary Table 1. Average IC_{50} values for tested compounds in $DU145^{WT}$and $DU145^{J7}$ using 48h luminescent cell viability assay. Cells were plated $9x10^3$ cells/well in a 96-well plate and tested using 10 different increasing concentrations of each agent. ($n \geq 2$) (*Experiment had two biological replicates; **Experiments had three biological replicates).

1.2 Supplementary Figure 1

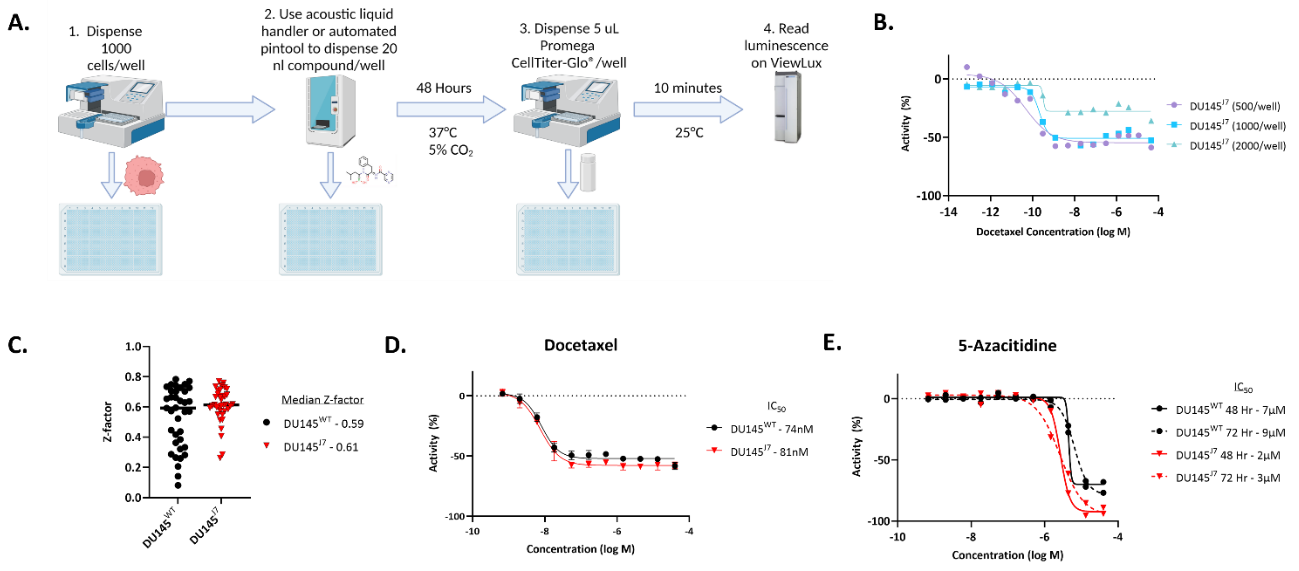

Supplemental Figure 1 A-E. Optimized 1536 well cell viability assay. (A) Schematic depicting the protocol for the optimized assay. Figure created in Biorender. **(B)** Optimization of DU145^{J7} cell density using a docetaxel titration. **(C)** Plot of the Z-factors for all plates in the primary screen. **(D)** Representative plot of the dose response data for docetaxel. The data are the average values of three technical replicates; error bars represent standard deviation for each data point. **(E)** Comparison of 48- and 72-hour treatment data for 5-azacitidine, one of the DU145^{J7} -selective compounds identified in the qHTS assay that was prioritized for additional study.

1.3 Supplementary Figure 2

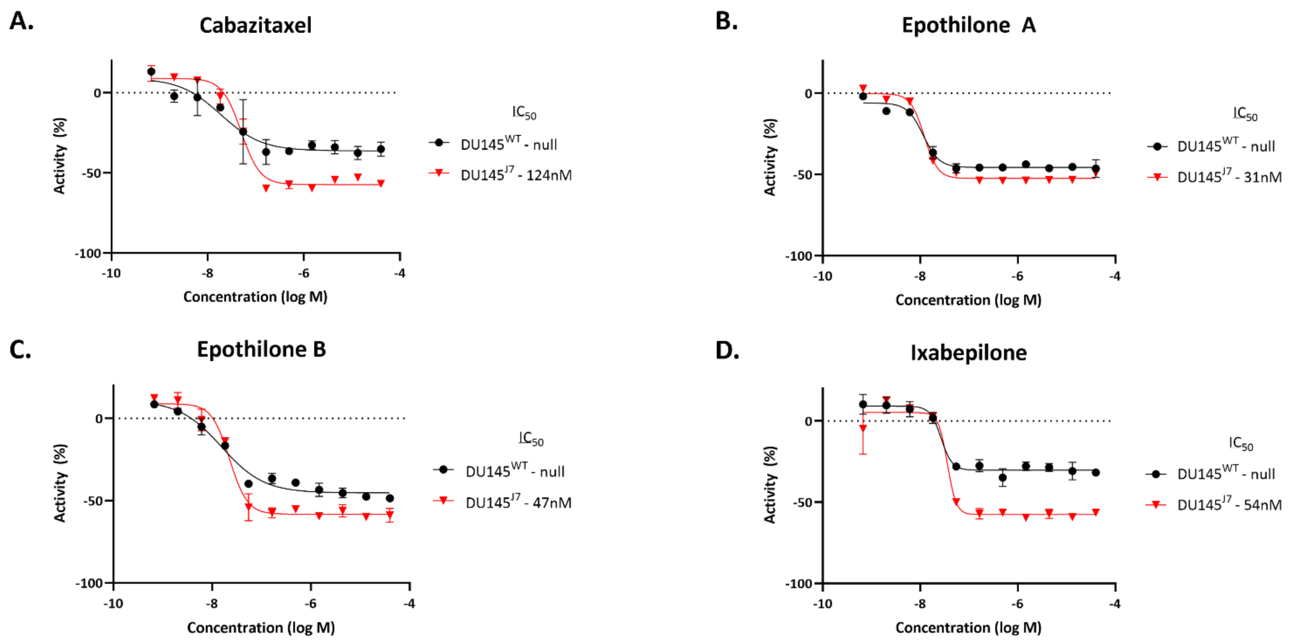

Supplemental Figure 2 A-D. Dose response plots for taxanes from qHTS. (**A-D**) Representative dose response plots for all taxanes present in the follow-up screen besides docetaxel. The data are the average of two technical replicates. Error bars represent standard deviation for each data point.

1.4 Supplementary Figure 3

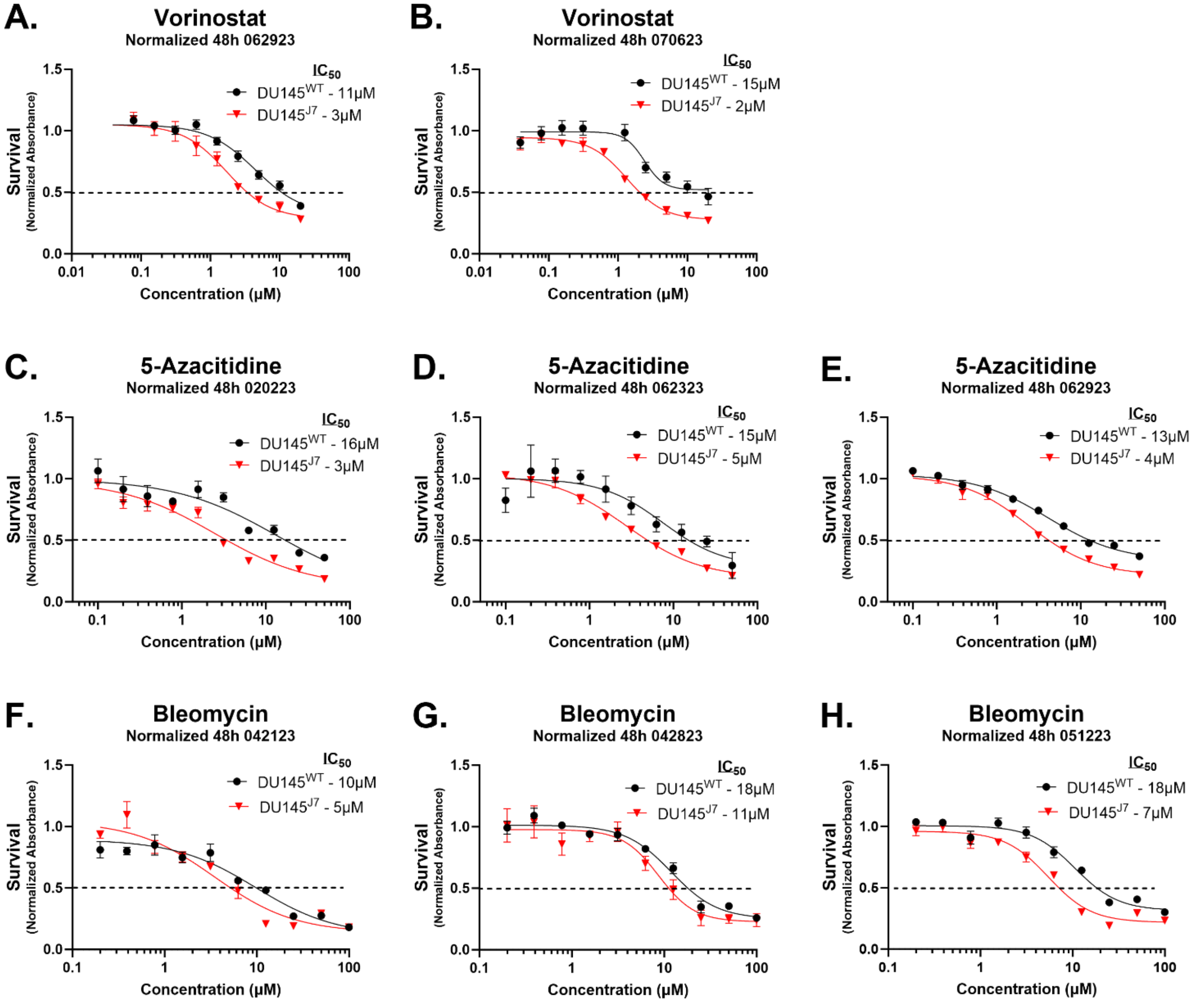

Supplemental Figure 3. Vorinostat, 5-Azacitidine, and Bleomycin Biological Replicates- Dose response curves of compounds demonstrating therapeutic vulnerability against aggressive $DU145^{J7}$ (red triangles) compared to $DU145^{WT}$ (black squares), *in vitro*. Points represent average values of technical replicates and error bars represent standard deviation for each data point.

1.5 Supplementary Figure 4

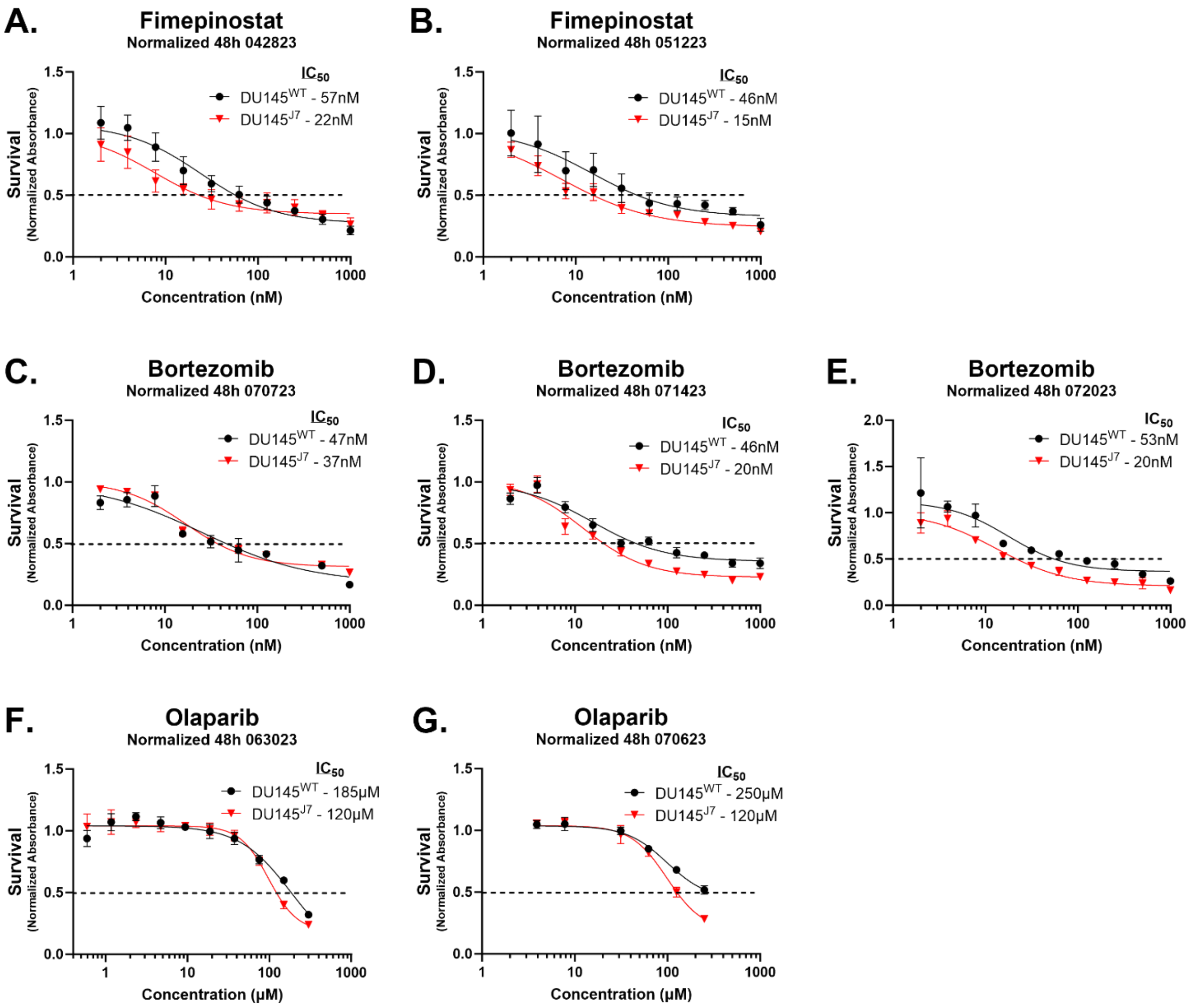

Supplemental Figure 4. Fimepinostat, Bortezomib, and Olaparib Biological Replicates- Dose response curves of compounds demonstrating therapeutic vulnerability against aggressive DU145^{J7} (red triangles) compared to DU145^{WT} (black squares), *in vitro*. Points represent average values of technical replicates and error bars represent standard deviation for each data point.

1.6 Supplementary Figure 5

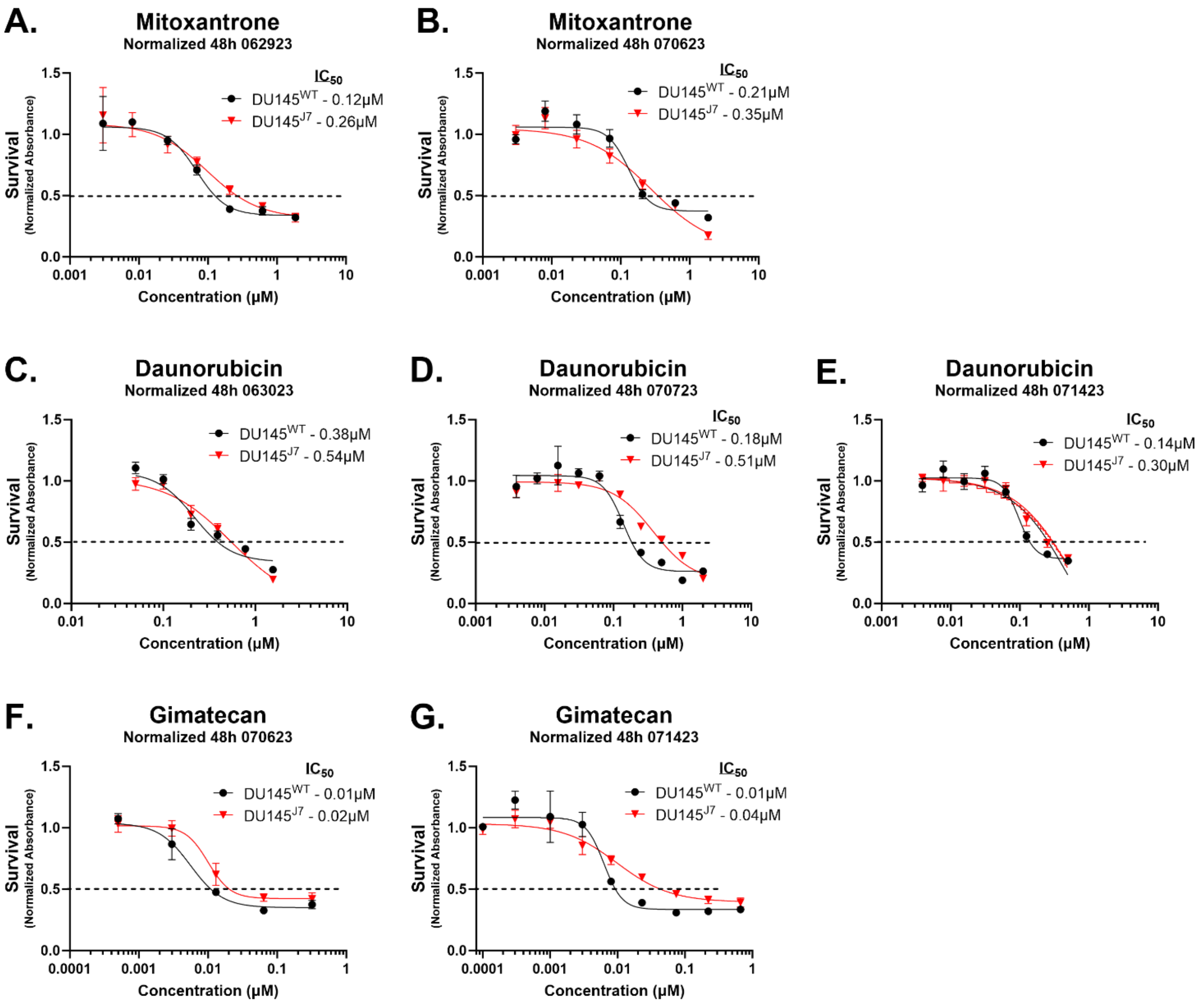

Supplemental Figure 5. Mitoxantone, Daunorucbicin, and Gimatecan Biological Replicates- Dose response curves of compounds demonstrating therapeutic vulnerability against aggressive DU145^{J7} (red triangles) compared to DU145^{WT} (black squares), *in vitro*. Points represent average values of technical replicates and error bars represent standard deviation for each data point.

1.7 Supplementary Figure 6

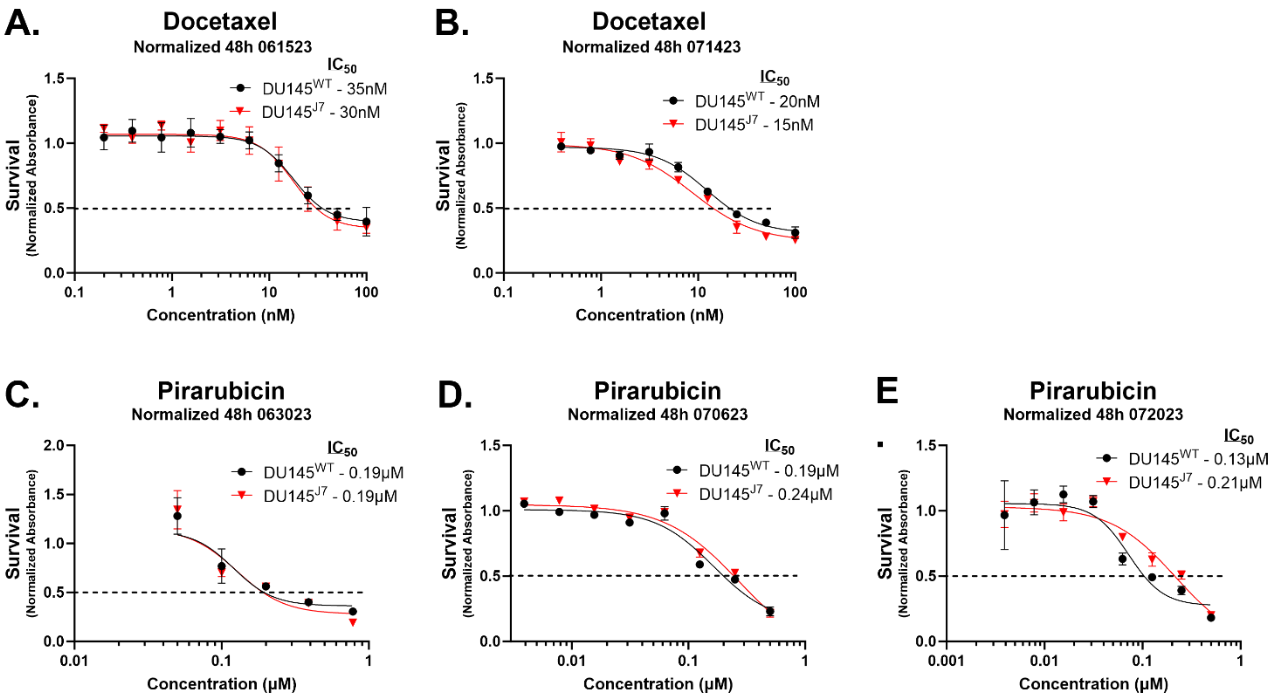

Supplemental Figure 6. Docetaxel and Pirarubicin Biological Replicates- Dose response curves of compounds demonstrating therapeutic vulnerability against aggressive $DU145^{J7}$ (red triangles) compared to $DU145^{WT}$ (black squares), *in vitro*. Points represent average values of technical replicates and error bars represent standard deviation for each data point.
